# How I do it: sling transposition technique with biopatch and aneurysm clip for hemifacial spasm

**DOI:** 10.1007/s00701-025-06634-0

**Published:** 2025-09-30

**Authors:** Sergio Corvino, Vincenzo Seneca, Giorgio Iaconetta, Giuseppe Catapano

**Affiliations:** 1 Department of Neurosurgery, Ospedale del Mare, 80147 Naples, Italy; 2 Department of Neurosurgery, AOU “San Giovanni di Dio e Ruggi D’Aragona”, 84131 Salerno, Italy

**Keywords:** Microvascular decompression, Hemifacial spasm, Trigeminal neuralgia, Sling transposition, Fat pad

## Abstract

**Background:**

Microvascular decompression (MVD) represents the only definitive and non-ablative treatment for hemifacial spasm (HS). Teflon is the most used interposing material because considered inert; nevertheless, it is not free from complications.

**Method:**

We discuss and illustrate our method to resolve the neurovascular conflict accounting for HS through a sling transposition technique using aneurysm clip and biopatch in carefully selected cases.

**Conclusion:**

Several MVD procedures, including interposing and transposing techniques, have been proposed for HS, mainly selected according to surgeon’s preference. We consider the described technique a definitive treatment, safe, not associated to pain recurrence and without needing revision surgery.

**Supplementary Information:**

The online version contains supplementary material available at 10.1007/s00701-025-06634-0.

## Relevant surgical anatomy

The relevant surgical anatomy includes all the main nervous and vascular structures of the cerebellopontine angle (CPA), organized into upper, middle and lower neurovascular complexes [5].

Despite the inestimable contribution provided by intraoperative tools, such as neuronavigation system, neurophysiological monitoring, fluorescein and indocyanine green (ICG) angiography, the deep knowledge of the microsurgical anatomy of this region rich of highly functional and vital neurovascular structures, as well as of the main tricks to identify them, is mandatory.

The first anatomical-surgical landmark to expose the CPA is the asterion, at the junction of the lambdoid, parietomastoid, and occipitomastoid sutures on the skull, and which can be identified on the skin at the junction between the horizontal line connecting the inion to the root of zygoma and the vertical line marking the mastoid groove hosting the posterior belly of digastric muscle. The drilling of the asterion reveals the inferior-medial aspect of the junction between transverse sinus and sigmoid sinus.

**Fig. 1:**
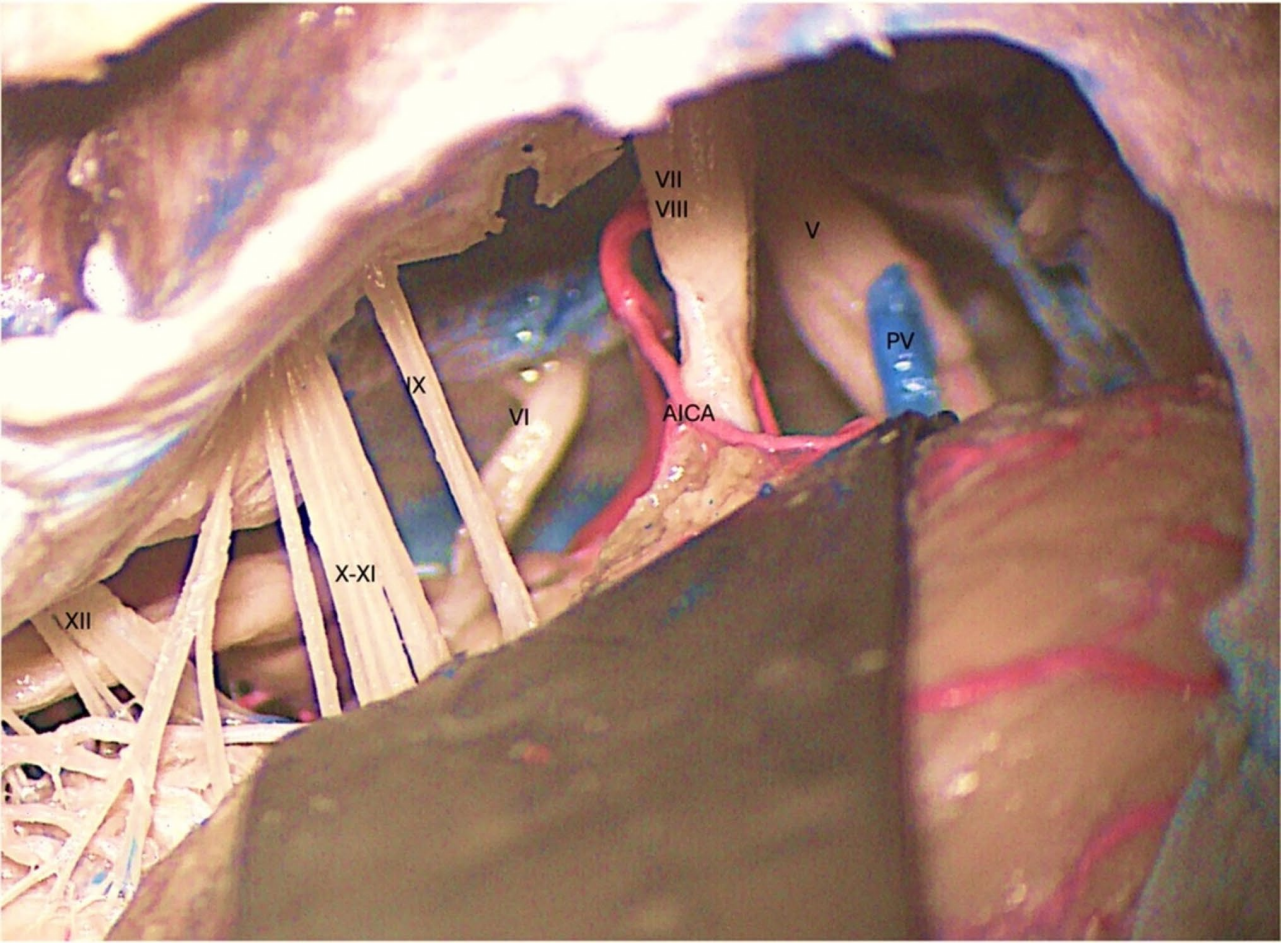
Retrosigmoid exposure of the left cerebellopontine angle in a cadaveric head specimen.(AICA: anterior inferior cerebellar artery; PV: petrosal vein)

The cerebellopontine angle is between the superior and inferior limbs of the cerebellopontine fissure formed by the petrosal cerebellar surface which folds around the pons and the middle cerebellar peduncle [5]. The fourth through the eleventh cranial nerves are near or within this area (Fig. 1).

The seventh cranial nerve arises from the brainstem at the lateral end of the ponto-meduallary sulcus, 1–2 mm medial to the vestibulocochlear nerve, anterosuperior to the choroid plexus protruding from the foramen of Luschka, anterior to the flocculus, and rostral to an imaginary line along the junction of the rootlets of the IX-X-XI with the brainstem.

The most common finding of neurovascular conflict involves the premeatal or postmeatal segments of the anterior inferior cerebellar artery (AICA) which comes into contact on the anterior-inferior aspect of facial nerve root entry zone (REZ) into the brainstem. Other potential offending vessels include a tortuous posterior inferior cerebellar artery (PICA) or vertebral artery (VA), a serpentine basilar artery (BA) and, less frequently, a combination of these vessels.

## Description of the technique

Intraoperative neurophysiological monitoring is routinely adopted for this procedure, including brainstem auditory evoked responses, trigeminal nerve somatosensory evoked potentials and facial motor evoked potentials. A standard retrosigmoid approach to the CPA was performed by the senior author (G.C.).

The patient is positioned in a three-quarter-prone position, with the head flexed and rotated contralaterally to the lesion.

A linear vertical incision crossing the asterion and 2 cm medial to mastoid process is performed. After a small craniectomy, roundish in shape and with a diameter about of 3 cm, on the asterion, the lower margin of the transverse sinus and the posterior margin of the sigmoid sinus are exposed, and the dura mater is opened in a cruciate fashion. Under microscopic magnification, the cerebellum is relaxed by opening the arachnoid of the dorsal cerebellomedullary cistern which covers the lower cranial nerves, and draining cerebrospinal fluid (CSF). Thus, the dissection is carried down on the cistern, the arachnoid behind the IX and X cranial nerves is opened, and the choroid plexus exiting from the Luschka foramen is identified. At this point, the seventh nerve could be hidden by the flocculus and the IX nerve. Therefore, to expose its entry zone into the brainstem, sometimes it is necessary to separate the choroid plexus from the glossopharyngeal nerve with a narrow brain spatula, usually 3 cm wide, which should be positioned parallel the sigmoid sinus on the petrosal surface of the cerebellum.

Once the VII cranial nerve is recognized, its course is inspected from the root entry zone at brainstem to the internal acoustic meatus on the petrous bone and its relationships with near anatomical structures are evaluated.

This stage represents an identification process and not a discovery, through a translation of the relevant neuroradiological anatomy carefully assessed during the preoperative planning through TOF (time of flight) or CISS (constructive interference in steady state) MRI sequences in the surgical filed [2]. The most common finding is a segment of the anterior inferior cerebellar artery (AICA), compressing the facial nerve on its anterior-inferior aspect, at its root entry zone in the brainstem. Once the neurovascular conflict is identified, a biological patch is harvested into a sling and is wrapped around the offending vessel at the contact point, paying attention to avoid a kinking of the vessel during transposition. Thus, the offending vessel is lifted away from the REZ and secured through an aneurysm clip to an anchor point created through an incision on the dura mater of the posterior surface of the petrous bone. ICG angiography is used to assess the patency and the absence of any kinking of the transposed AICA.

After verifying the nerve is free from any type of compression, dural edges are closed and the muscles and the skin are approximated in two different layers.

Early postoperative management after MVD for HS focuses on gradual activity resumption, pain management, and monitoring for potential complications. Antiemetics, analgesics and intravenous fluids are administered for 24 h as postoperative transient, vertigo and ataxia, and vomiting may occur. Patient is usually discharged on the third or fourth postoperative day, while stitches are removed on the eighth or ninth postoperative day.

### Illustrative case

A 67-year-old woman was observed for a 2-years history of left facial spasms involving the superior eyelid and orbicularis oculi muscle that caused involuntary and intermittent homolateral eye closure and initially treated with serial botulinum toxin injections but with transient and limited benefits. Few months after the clinical onset, the spasms became more frequent and extended to involve the larger buccal and orbicularis oris muscles and were not controlled even after higher-dose injections.

**Fig. 2:**
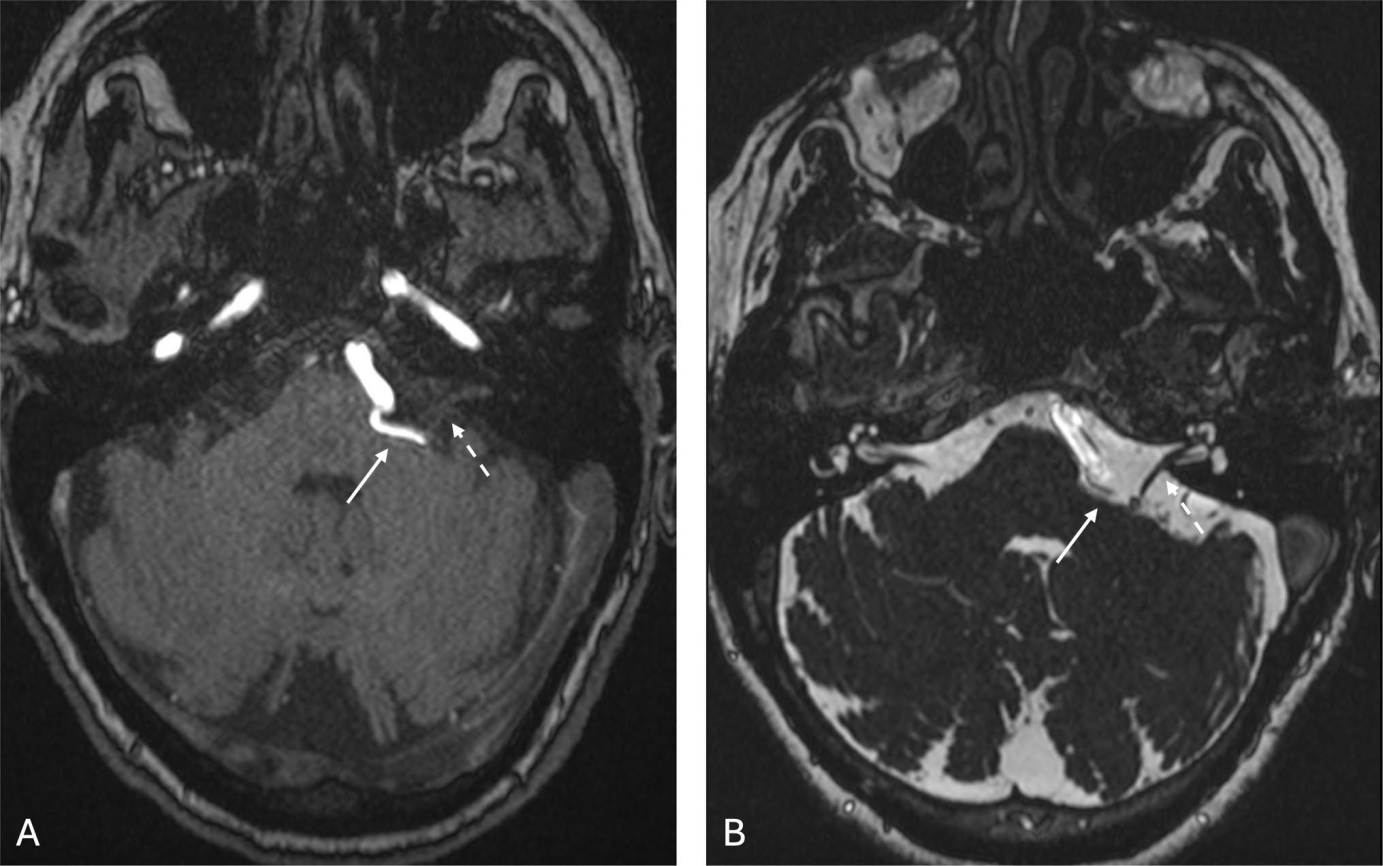
Preoperative MRI of the brain: TOF (**A**) and CISS (**B**) sequences. A segment of the Anterior inferior cerebellar artery (white arrow) compresses the facial nerve (white dotted arrow) in the left cerebellopontine angle

TOF (Fig. 2A) and CISS (Fig. 2B) Angio-MRI scans showed the culprit neurovascular conflict between the postmeatal segment of AICA and the inferior surface of facial nerve on the left CPA. Because of the clinical manifestations and neuroradiological picture, a surgical option was offered to the patient.

**Fig. 3:**
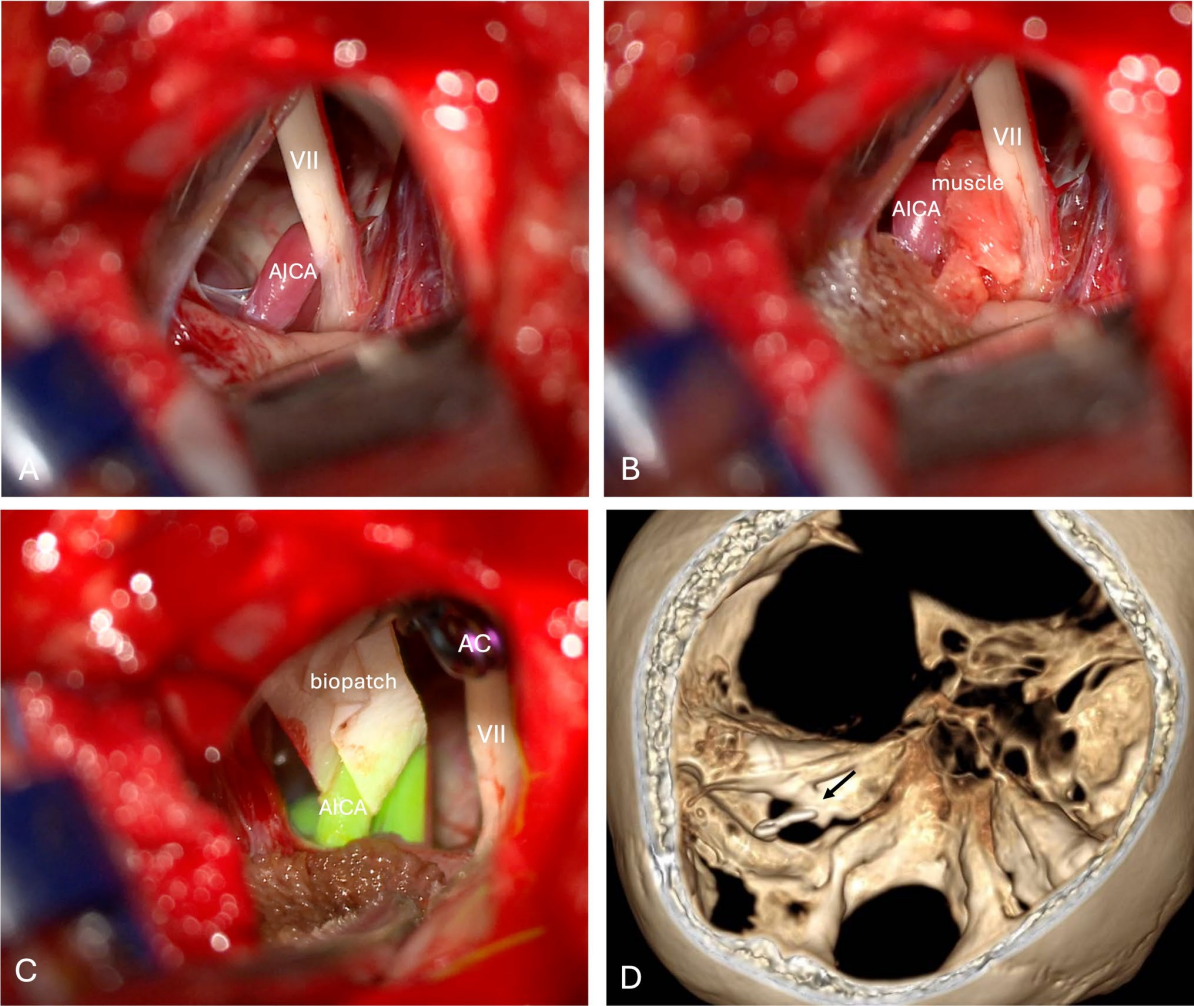
Intraoperative view. **A**) Neurovascular conflict between premeatal segment of AICA and facial nerve; **B**) Microvascular decompression with interposition of free graft of autologous muscle; **C**) Sling transposition technique of the offending vessel with biopatch anchored to the dura of posterior surface of the petrous bone with aneurysm clip and control with indocyanine green angiography. **D**) CT scan 3D reconstruction: aneurysm clip anchored to the dura of posterior surface of the petrous bone (AICA: anterior inferior cerebellar artery; AC: aneurysm clip)

**Fig. 4:**
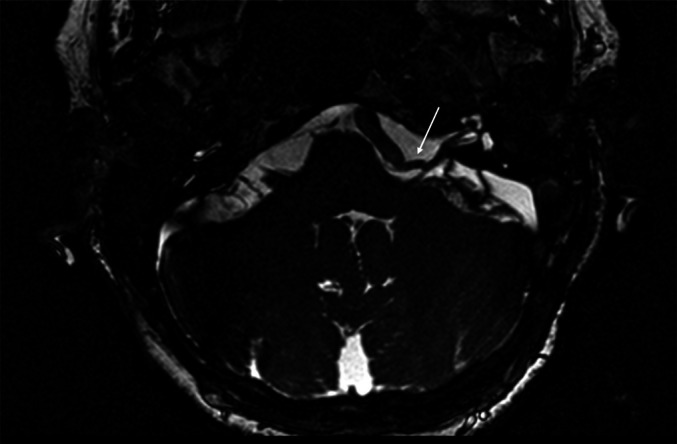
Postoperative MRI of the brain. Resolution of the neurovascular conflict in the left cerebellopontine angle between AICA (white arrow) and facial nerve at its REZ after transposition of the offending vessel

Initially we attempted to perform a classic MVD with interposition of a free graft of autologous muscle between the compressed nerve and the offending vessel, but the neurophysiological monitoring immediately registered a sufferance of the nerve even with a small piece of muscle as pad. In addition, the persistence of pulsation transmission on the nerve was also observed (Vid.1). Therefore, we opted for the sling transposition technique (Vid.1 & Fig. 3). Intraoperative ICG angiography confirmed the patency and the absence of any kinking of the transposed AICA (Vid 1.). Postoperative MRI confirmed the neurovascular conflict resolution and the patency of the transposed vessel (Fig. 4).

Patient referred a resolution of the hemifacial spasms in the follow-up.

## Indications

MVD is the treatment of choice for HS refractory to high dose and serial injections of botox with evidence of a neurovascular conflict on MRI [3]. This procedure includes two techniques: transposition of the offending vessel from the compressed cranial nerve and the interposition of a pad between the nerve and the blood vessel. Despite the rationale is always the same—to alleviate pulsatile vascular compression on the facial nerve—both methods include several variations in terms of materials adopted and surgical technique, each with related pros and cons [6]; the course and anatomy of the offending vessel and the way it creates the conflict is the main criterion for the technique selection. For instance, direct vertebral artery conflict, short course of the culprit vessel, evidence of microperforators with short course in the proximity of the conflict zone will hamper the opportunity of transposition and will orient toward interposition. Nevertheless, there are not well-defined guidelines for the selection of the optimal technique, and the decision is usually demanded to the surgeon’s preference.

In our opinion, the sling transposition technique with aneurysm clip is useful when large volumes of interposing material are required to separate the offending vessel and nerve because of the close and long relationship of the natural course of the vessel and nerve or in presence of a large culprit vessel providing intense pulsations which could account for pad displacement or persisting facial pain, thus potentially requiring revision surgery. Particularly, it is indicated when the interposing material results in a sufferance of the nerve confirmed by the intraoperative neurophysiological monitoring.

Conversely from the interposition technique which can be associated to pain recurrence for conditions materials-related (granuloma, adherences, displacement, resorption) and thus to revision-surgery, the main advantage of the present technique is its definitive treatment.

## Limitations

The transposition technique through aneurysm clip requires expertise in neurovascular surgery. Neurosurgeon must be confident with aneurysm clipping technique.

## How to avoid complications

The relevant regional anatomy of the cerebellopontine angle and the middle neurovascular complex, including the neurovascular conflict and the adjacent vessels and nerves, must be clearly recognized through a small craniectomy. The common cause of failure is a misdirected surgery [1], directed at the cisternal portion of the facial nerve, while the culprit compression at the more proximal REZ had not been inspected or decompressed; therefore, it is crucial to expose and inspect the root entry zone of the facial nerve into the brainstem. Attention must be paid to avoid the kinking of the offending vessel during its transposition, which can be assessed by direct visualization, with intraoperative Doppler flow probe, and with fluorescein or green indocyanine angiogrphy. Furthermore, the surgeon must be sure that the distal clip blades do not enclose any surrounding vessels, whose patency must be ensured and intraoperatively verified.

## Specific information to give to the patient about surgery and potential risks

Patients should be informed about the rationale for the treatment and existing alternatives, with relates pros and cons, mainly the rate of long-term pain relief. Interposition techniques are associated to higher rates of earlier spasm resolution but higher rates of recurrence [4]. Major risks associated to this procedure include intraoperative hemorrhage and postoperative ischemia, facial nerve palsy, hearing loss, cerebrospinal fluid leakage.

## Ten key points

1. The most common etiology of hemifacial spasm is represented by a neurovascular conflict between the anterior inferior cerebellar artery and facial nerve at its root entry zone in the brainstem.
2. Two different techniques but with the same rationale are usually performed: transposition of the offending vessel from the facial nerve root entry zone and the interposition of a pad between the nerve and the blood vessel.
3. Despite MVD through interposition technique provides high rates of earlier spasm resolution, it can be associated to recurrence and necessity of revision surgery for complications related to the pad. The vessel transposition with aneurysm clip anchored to the dura mater represents a definitive cure for HS, avoiding risks of revision surgery.
4. The common cause of failure is surgery directed at the cisternal portion of the facial nerve, while the culprit compression at the more proximal REZ had not been inspected or decompressed; therefore, it is very important intraoperatively inspect the facial nerve at the root entry zone into the brainstem.
5. The anteroinferior trajectory to the lower cranial nerves is preferable to gain access to the facial nerve REZ over a lateral approach directed to the internal acoustic meatus and cisternal portion of its associated nerves.
6. Patients with large offending vessel, for a long curse in close relationship with the facial nerve, requiring large amount of interposing material, represent the optimal candidate for sling transposition technique.
7. An initial MVD with an interposing pad can be attempted; the response of the neurophysiological monitoring and the direct visualization assist the surgeon in the selection of the technique, shifting toward the sling transposition technique.
8. The sling must be meticulously harvested in shape and size to avoid the kinking of the transposed vessel.
9. An important trick is to start mobilization of the offending vessel away from the point of maximum compression.
10. The use of an aneurysm clip to hold the sling to a dural anchor point is easy to perform and avoid the maneuvers of suturing in a narrow space.
11. A careful intraoperative assessment of the patency of the transposed vessel, as well as of any surrounding vessel, is mandatory

## Supplementary Information

Below is the link to the electronic supplementary material.

ESM 1 (MP4 401 MB)

## Data Availability

No datasets were generated or analysed during the current study.
